# Artificial Neural Network Model with Astrocyte-Driven Short-Term Memory

**DOI:** 10.3390/biomimetics8050422

**Published:** 2023-09-12

**Authors:** Ilya A. Zimin, Victor B. Kazantsev, Sergey V. Stasenko

**Affiliations:** 1 Laboratory of Advanced Methods for High-Dimensional Data Analysis, Lobachevsky State University of Nizhny Novgorod, 603022 Nizhny Novgorod, Russia; izeemeen@gmail.com (I.A.Z.); kazantsev@neuro.nnov.ru (V.B.K.); 2 Laboratory of Neurobiomorphic Technologies, Moscow Institute of Physics and Technology, 117303 Moscow, Russia

**Keywords:** short-term memory, convolutional neural network, machine learning, neuron–glial interaction

## Abstract

In this study, we introduce an innovative hybrid artificial neural network model incorporating astrocyte-driven short-term memory. The model combines a convolutional neural network with dynamic models of short-term synaptic plasticity and astrocytic modulation of synaptic transmission. The model’s performance was evaluated using simulated data from visual change detection experiments conducted on mice. Comparisons were made between the proposed model, a recurrent neural network simulating short-term memory based on sustained neural activity, and a feedforward neural network with short-term synaptic depression (STPNet) trained to achieve the same performance level as the mice. The results revealed that incorporating astrocytic modulation of synaptic transmission enhanced the model’s performance.

## 1. Introduction

Short-term memory, also referred to as working memory, is a fundamental cognitive process crucial for temporary storage and manipulation of information. It plays an important role in attention, learning, problem-solving, and decision-making. The multi-component model proposed by Baddeley and Hitch [1] suggested that short-term memory consists of the phonological loop, visuospatial sketchpad, and central executive. Empirical studies, such as Miller’s research [2], supported the concept of limited capacity in short-term memory, which can be understood in terms of “working memory chunks” [3]. Neuroimaging studies employing fMRI identified brain regions such as the dorsolateral prefrontal cortex, parietal cortex, and posterior regions involved in short-term memory [4]. Various factors such as interference, time, and individual differences can influence short-term memory performance [5,6,7].

Recent experimental and computational research indicated that persistent neural activity, facilitated by local recurrent connections or cortical–subcortical loops, was capable of storing information [8,9,10]. Sustained and sequential persistent activity has been observed in various tasks and brain regions, including the prefrontal cortex (PFC) [11]. Another mechanism for maintaining short-term memories involves short-term synaptic facilitation, utilizing residual calcium as a memory buffer [12,13]. However, recent experiments indicated that excitatory synapses in early sensory areas, such as the mouse primary visual cortex, primarily undergo synaptic depression [14]. This synaptic depression influenced visual processing by providing temporal context to distinguish between familiar and novel stimuli [14]. Additionally, adaptation at the synaptic level and intrinsic firing rate adaptation [15,16] may play a significant role in brain functioning.

Visual change detection tasks were extensively employed in cognitive psychology and neuroscience to investigate short-term memory for visual information. In these tasks, participants were presented with a set of visual stimuli that briefly disappear, and one or more stimuli undergo changes. Participants must detect and identify the item(s) changed. This paradigm was utilized in numerous studies exploring various aspects of short-term memory, including capacity, encoding processes, and the effects of attention. For example, Luck and Vogel [17] found that participants could reliably remember and detect changes in approximately three to four objects in a display. Hollingworth and Henderson [18] demonstrated a crucial role of attention in maintaining and updating visual information in short-term memory. Neuroimaging studies utilized visual change detection tasks to investigate the neural mechanisms underlying short-term memory, with Vogel et al. [19] using electroencephalography (EEG) to examine the neural correlates of visual change detection. Visual change detection tasks have provided valuable insights into the capacity, attentional processes, and neural mechanisms involved in visual short-term memory.

Astrocytes, traditionally considered as supportive cells in the brain, have emerged as active participants in various brain functions, including memory processes. Recent research suggested that astrocytes play a role in modulating short-term memory [20]. Various studies demonstrated their involvement in regulating synaptic transmission [21], promoting metabolic interactions for long-term memory formation [22], and organizing inhibitory circuits in the cerebellum [23]. These findings indicated that astrocytes exert influence on short-term memory processes participating in the complex dynamics of memory formation and maintenance.

Convolutional neural networks (CNNs) are a type of deep learning architecture specifically designed for processing grid-like data, such as images and videos. CNNs are inspired by the visual processing mechanism of the human brain and are particularly effective in tasks such as image recognition, object detection, and image segmentation [24]. The applications of CNN are extremely wide and include medical data analysis [25,26], autonomous vehicles [27], natural language processing [28], image style transfer [29], computational chemistry [30], and environmental monitoring control systems [31]. There is no direct relationship between CNNs and short-term memory (STM) in the cognitive sense. CNNs do not have memory components that function as human STM. However, recurrent neural networks (RNNs) [24] are a different type of neural network architecture that is often used for tasks involving sequential data and can exhibit memory-like behavior. Unlike CNNs, RNNs have internal memory cells that can store and process information across time steps, which makes them suitable for tasks where the order and context of data matter, such as natural language processing. Adding memory-like behavior to CNNs can expand the possibilities of their applications.

This study proposes a novel hybrid model of short-term memory that incorporates short-term synaptic plasticity, astrocytic modulation of synaptic transmission, and a convolutional neural network. When compared to the recurrent neural network, the proposed model demonstrates better efficiency in the implementation of short-term memory.

The structure of the paper is as follows. Section 2 outlines the mathematical model and methodology employed in this investigation. In Section 3, we present the primary findings from our examination of the model, both with and without the influence of astrocytic modulation on neuronal activity. We also compare our model to a recurrent neural network and experimental data, demonstrating that incorporating astrocytic modulation yields better alignment with the experimental results. Section 4 engages in a discussion of the obtained results and proposes potential avenues for further research. Finally, Section 5 provides a concise summary of the study’s outcomes.

## 2. Materials and Methods

**Figure 1:**
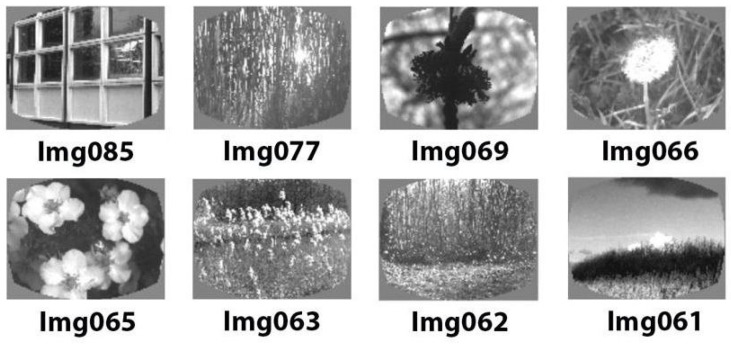
Sample of 8 images used for training.

We used experimental data obtained from mice during the visual change detection task using natural “Go”/“Catch” images, as described in the study by Hu et al. [32]. In that paper, the mice underwent a multi-step learning process, progressing from static bars to flashing bars, and eventually to natural images [33]. Spherically deformed stimuli were employed to account for variations in the distance from the eye to the monitor periphery (undistorted stimuli were used for simplicity in visualization). A set of eight contrast-normalized natural images, all with the same average brightness, was presented in grayscale (Figure 1). All figures were taken from the CIFAR-10 dataset.

**Figure 2:**
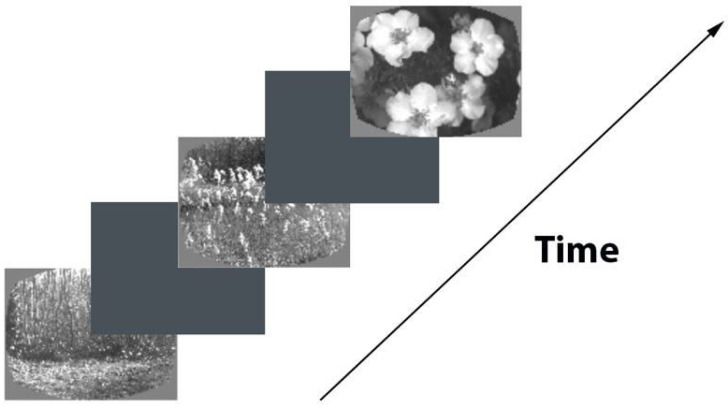
Scheme of image recognition task.

During the training phase, the mice were trained to drink water whenever a change occurred in the presented picture sequence. Each image was shown for 250 ms, followed by a 500 ms inter-stimulus interval with a medium gray brightness (see Figure 2). A small percentage (5%) of image presentations were randomly omitted, ensuring that these omissions never happened before an actual change in the image (referred to as “Go” trials) or a fictitious change in the image (referred to as “Catch” trials). Importantly, these omissions were only present during visualization sessions and not during the actual training sessions. Correct responses were rewarded with water, while premature licking triggered a “time-out” period during which the probe logic timer was reset. In “Go” trials, the images were switched, and the mice had to indicate the change to receive a water reward. Conversely, “Catch” trials involved unchanged images and were used to measure false alarms.

### 2.1. Dynamic Synapse Model

In this study, we employed an approach described in several references [34,35,36] to investigate the dynamics of astrocytes, which are a type of glial cell. In this context, the activation of excitatory neurons triggers the release of neurotransmitters, represented by the variable x(t), with the probability of neurotransmitter release denoted as *u*. Subsequently, when the neurotransmitter binds to receptors on the astrocyte membrane, a series of biochemical reactions occur, leading to the release of gliotransmitters. The dynamics of gliotransmitters are captured by the variable y(t). As a result, the proposed model can be formulated as a system of ordinary differential equations (ODEs).

(1) $$\left\{\begin{array}{c} \frac{dx}{dt}=\frac{1-x}{\tau_{D}}-ux\tau \left(t_{i}\right),\\ \frac{dy}{dt}=\frac{-y}{\tau_{y}}+\beta H_{y}\left(x\right), \end{array}\right.$$

(2) $$H_{y}\left(x\right)=\frac{1}{1+e^{-20(x-x_{thr})}}$$

(3) $$u\left(y\right)=u_{0}+\frac{\Delta u_{0}}{1+e^{-50(y-y_{thr})}}$$

In Equation (1), the variable $\tau_{D}$ represents the time constant governing synaptic depression. The term τ(t) signifies the presynaptic activity at time $t_{i}$, and $\tau_{y}$ corresponds to the relaxation time of the gliotransmitter. The activation function $H_{y}\left(x\right)$ is determined by Equation (2), and $x_{thr}$ denotes the activation threshold.

As a dynamic synapse model (Equation (1)), we employ a mean-field model based on the Wilson–Cowan formalism [37] (which does not consider spiking dynamics but focuses on averaged neuronal activity) along with the Tsodyks–Markram model [38]. In this context, presynaptic activity, τ(t), essentially reflects the average neuronal activity, which, within our framework, can be described as follows:

(4) $$\tau \left(t_{i}\right)=n\times {out^{L}}_{i}.$$

**Figure 3:**
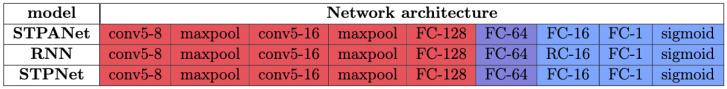
Model STPNet includes only short-term synaptic adaptation, whereas STPANet also contains astrocytic regulation in modulating synaptic dynamics. RNN refers to a recurrent neural network. The convolutional layers are denoted as “conv<receptive field size>-<number of channels>”. The term “maxpool” indicates the use of max pooling with a 2 × 2 window and a stride of 2. “FC” represents fully connected layers with a specified number of units, while “RC” signifies recurrent layers with a specified number of units.

In Equation (4), the variables are interpreted as follows: τ(t) represents a vector denoting presynaptic activity at distinct time points $t_{i}$. Each element of the vector corresponds to a specific time point. The variable *n* signifies the total number of experiments conducted. ${out^{L}}_{i}$ refers to the activation function of the *i*-th element within the FC-64 layer of the convolutional neural network (depicted in Figure 3). Equation (4) describes how τ(t) changes over time, influenced by the number of experiments conducted (*n*) and the output from the FC-64 layer of the CNN ($out^{L}$).

The activity of astrocytes leads to the release of gliotransmitter, which, upon binding to the membrane receptors of the presynaptic neuron, modulates the probability of neurotransmitter release according to Equation (3). In this context, u(y) represents the influence of astrocytes on the likelihood of glutamate release from the presynaptic neuron. Additionally, $u_{0}$ denotes the probability of neurotransmitter release in the absence of glial interaction, $\Delta u_{0}$ signifies the change in release probability influenced by astrocytes, and $y_{thr}$ represents the activation threshold.

We adopted the standard parameters from the Tsodyks–Markram model [38], which are widely used to characterize neural activity. For the neurotransmitter and gliotransmitter parameters, we followed the tripartite synapse model proposed in previous studies [36,39]. Specifically, we set $\tau_{D}$ to 6. The values for the neurotransmitter and gliotransmitter parameters were the following: $u_{0}$ = 0.23, $\Delta u_{0}$ = 0.305, $\tau_{y}$ = 1.8, β = 0.4375, $x_{thr}$ = 0.5, and $y_{thr}$ = 0.573.

### 2.2. Connection of the Dynamic Synapse Model with an Artificial Neural Network

Differential Equations (1)–(3) are used in this model, including astrocytic regulation, to describe the dynamics of chemical concentrations and their interactions in a neural network. In this model, diffuse equations describe the distribution of neuromodulators that act as mediators between neurons and astrocytes.

**Figure 4:**
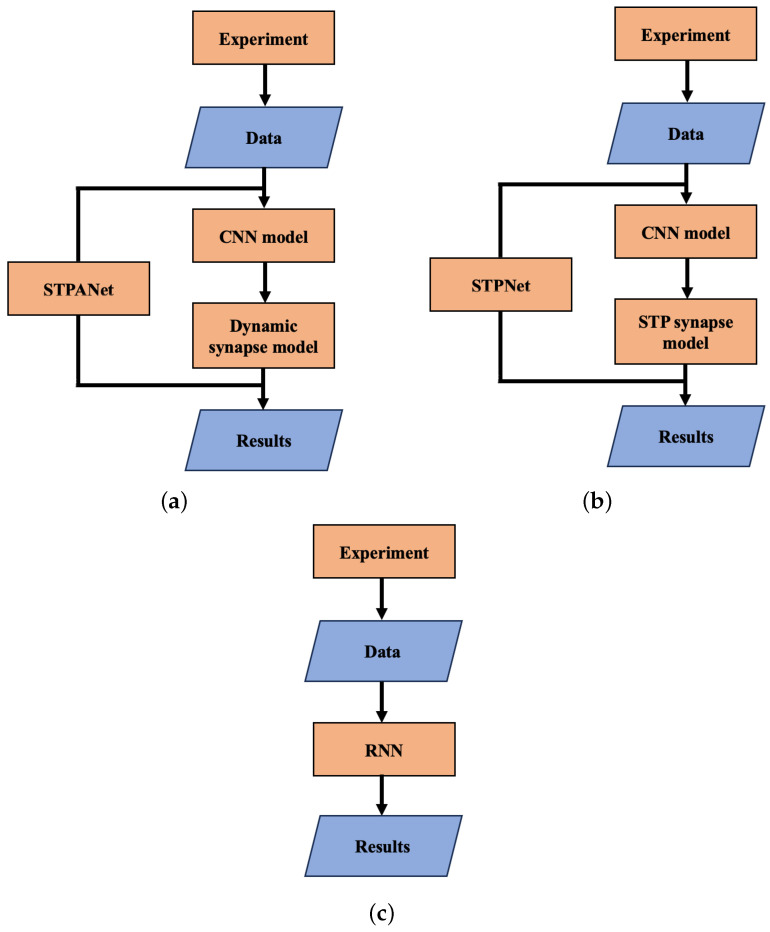
Block diagram of the work carried out. (**a**) STPANet, (**b**) STPNet, (**c**) RNN.

As can be seen in Figure 4a, the STPANet model consists of two submodels: a CNN and a dynamic synapse model. The output layer with CNN Equation (14) with stored weights plays the role of presynaptic activity τ(t) Equation (1). Further, according to Algorithm 1, we train a dynamic synapse model, which consists of three layers: an input layer with 64 neurons, a hidden layer with 16 neurons, and an output neuron. The model weights were trained using backpropagation. Equations (1)–(3) are used in the conversion step between layers of 64 neurons and 16 neurons. We emphasize that this plasticity is presynaptic, but it can interact with the weight updates from backpropagation during neural network training.
**Algorithm 1** Learning algorithm for dynamic synapse model**Input:** matrix of preprocessed input objects, initial distribution of weights, neuron parameters, plasticity parameters, vector of classes.**Parameter:** N_epochs, Adam optimizer, BSE loss function, threshold, patience.**Output:** distribution of weights of the neural network, spike times of the output.1.Initialize a stimulus generator and define the input dimension of the feature vector.2.Create a neural network model based on the dynamic synapse model.3.Define the loss function and optimizer.4.Initialize tracking variables for loss and d-prime.5.**for** in N_epochs **do**6.   Generate train batch.7.   Execute a forward pass within the model.8.   Compute the loss function by comparing the predicted values with the target.9.   Perform backpropagation.10.**end for**11.Append the loss and d-prime values to the corresponding lists for tracking.12.**if** d−prime<threshold13.    Reset the wait_count.14.**else**15.    Increase the wait_count.16.    **if** wait_count≥patience17.           Break the training loop18.     **end if**19.**end if**20.Finish the algorithm execution.

By incorporating differential equations, the STPANet model captures the temporal dynamics and spatial distribution of chemical substances, enabling a more comprehensive understanding of how neuromodulators propagate and affect neural activity.

In summary, the inclusion of differential equations, along with astrocytic regulation, in the STPANet model provides a more comprehensive framework for studying the dynamics of chemical interactions within neural networks. These equations enable the modeling of neuromodulator diffusion, synaptic plasticity, and the impact of astrocytes, contributing to a deeper understanding of the complex mechanisms underlying neural network behavior and plasticity.

### 2.3. Numerical Simulation Method

Numerical integration was performed using the Euler method. The implementation of numerical methods and data analysis utilized the Python [40] programming language, along with the PyTorch library [41] for model simulation, NumPy [42] for arrays, and the Matplotlib library [43] for data visualization and analysis. As a discretization step, a value was obtained, which is derived from this equation:

(5) $$t=\frac{length}{time\_step}$$

where length is the duration of each experiment in milliseconds = 50,000 ms and time_step is simulation time step, which is numerically equal to the time one image is shown = 250 ms. Therefore, we compare models in the range of 200 conventional units, where 1 conventional unit corresponds to the sampling step, i.e., the time of displaying one image.

### 2.4. D-Prime Metric

To evaluate the efficacy of our model compared to a neural network architecture that incorporates only short-term synaptic depression and a recurrent network, we utilized the d-prime metric (detectability index). A higher value of this index indicates better signal recognition. This metric, originally introduced in the study by Hu et al. [32], was employed to assess the capacity to retain and recall a sequence of images.

The d-prime index is calculated using the following formula:

(6) $$d=\frac{\left(\mu (hit\_rate)-\mu (false\_rate)\right)}{\sigma }$$

where σ is the standard deviation and μ(hit_rate) and μ(false_rate) are “Go” and “Catch” trial distributions, respectively.

Distributions of “Go” and “Catch” are obtained in several stages:

1. A sigmoid activation function is fed to the output layer from the model so that the output takes values strictly from 0 to 1:
  (7) $$S\left(\omega \right)=\frac{1}{1+e^{-\omega }}$$
  where ω is an array of the “Go” values or “Catch” values.
2. After applying the activation function, draw binary random numbers (0 or 1) from a Bernoulli distribution. The *i*-th element of the prediction will draw a value according to the *i*-th probability value given in output:
  (8) $$prediction_{i}∼Bernoulli\left(p=output_{i}\right)$$
3. Introduce the notation for “Go” and “Catch” experiments:
  “Go”: labels≡1
  “Catch”: labels≡−1
  After that, we can evaluate μ(hit_rate) and μ(false_rate):
  (9) $$\mu \left(hit\_rate\right)=\frac{\sum (prediction\times (labels\equiv 1))}{\sum (labels\equiv 1)}$$
  (10) $$\mu \left(false\_rate\right)=\frac{\sum (prediction\times (labels\equiv -1))}{\sum (labels\equiv -1)}$$

### 2.5. Calculate Matrix Asymmetry

The metric utilized is the ratio of the difference between the symmetric and anti-symmetric matrices to the sum of their norms. The formula for this metric is as follows:

(11) $$Q=\frac{\left|\right|M_{sym}-M_{antisym}\left|\right|}{\left|\right|M_{sym}-M_{antisym}\left|\right|}$$

- Compute the symmetric matrix ($M_{sym}$) by taking the average of the input matrix and its transpose:
  (12) $$M_{sym}=\frac{1}{2}\times \left(Matrix+Matrix.T\right)$$
- Compute the anti-symmetric matrix ($M_{antisym}$) by taking the difference between the input matrix and its transpose divided by 2:
  (13) $$M_{antisym}=\frac{1}{2}\times \left(Matrix-Matrix.T\right)$$

The resulting asymmetry metric (Q) provides a measure of the asymmetry between the symmetric and anti-symmetric components of the matrix. A higher value indicates greater asymmetry, while a value close to zero suggests a more balanced or symmetric matrix.

## 3. Results

In general, the work can be divided into several parts, as presented in the block diagram below (Figure 4).

In study [32], an experiment was conducted from which we obtained data for further research. Subsequently, two neural network models were constructed: a convolutional neural network and a dynamic synapse model. Based on the predictions obtained from these models, the results were summarized.

In this paper, we study the STPANet model, which consists of a convolutional neural network (CNN) model and a dynamic synapse model. The dynamic synapse model, Equations (1)–(3), is a short-term memory model with the addition of astrocytic regulation.

### 3.1. Hybrid Artificial Neural Network Model

**Figure 5:**
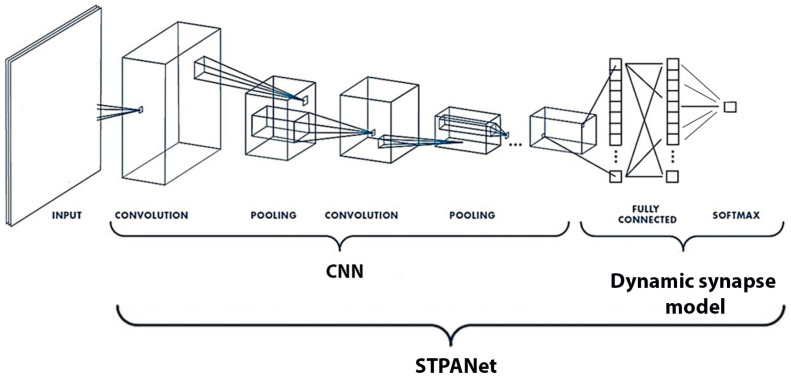
CNN: Image features were extracted from the last fully connected layer of a pre-trained convolutional neural network (CNN) trained on a grayscale version of the CIFAR-10 object recognition task. This CNN serves as an encoder network, mapping the input image to a lower-dimensional feature space, which is used as input data for the model. Dynamic synapse model: The model represents a neuron–glia network with short-term synaptic plasticity. It consists of three layers: an input layer with 64 neurons, a hidden layer with 16 neurons, and an output neuron. The model weights were trained using backpropagation.

A feed-forward neural network was utilized to encode a collection of natural images into a lower-dimensional feature space (see Figure 3 and Figure 5). The convolutional neural network (CNN) underwent training using a grayscale version of the CIFAR-10 dataset [44] and consisted of two convolutional layers followed by two fully connected layers. Once the training process was completed, the weights of the network were saved. For the dynamic synapse model, the input data were obtained by extracting the output of the last fully connected layer, which precedes the classifier.

The STPNet model incorporates only short-term synaptic adaptation, while the STPANet model includes additional astrocytic regulation for modulating synaptic dynamics. The RNN abbreviation stands for recurrent neural network, and the convolutional layers are labeled as “conv<receptive field size>-<number of channels>”. The term “maxpool” indicates the utilization of max pooling with a 2 × 2 window and a stride of 2. “FC” denotes fully connected layers with a specific number of units, while “RC” represents recurrent layers with a specified number of units. At the output of the convolutional neural network, we have a layer of size 64, all weights are saved, and in this form the data are fed to the input of one of three models: STPANet, RNN, STPNet.

CNN: In order to obtain image features, a pre-trained convolutional neural network (CNN) was utilized, which had been trained on a grayscale variation of the CIFAR-10 object recognition task. This CNN functioned as an encoder network, responsible for mapping the input image to a lower-dimensional feature space. These extracted features were then employed as input data for the subsequent stages of the model. By leveraging the pre-trained CNN’s learned representations, the model could effectively capture and utilize the rich visual information present in the images, enhancing its overall performance.

Dynamic synapse model: The architecture of the model encompasses a neuron–glia network that incorporates short-term synaptic plasticity. It comprises 3 distinct layers, starting with an input layer comprising 64 neurons, followed by a hidden layer housing 16 neurons, and culminating in an output neuron. The model’s weights were trained using the backpropagation algorithm, which facilitated the iterative adjustment of the weights based on the computed error signals. This training process enabled the model to learn and adapt its synaptic connections, optimizing its ability to process and generate accurate outputs. The utilization of short-term synaptic plasticity within the model allows for the dynamic modulation of synaptic strength, leading to more sophisticated information processing capabilities and enhancing the model’s overall performance.

The dynamic synapse model received an input represented by a matrix denoted as *M*:

(14) M(τ(t)×s).

In Equation (14), the variables have the following interpretations: τ(t) presynaptic activity and *s* indicate the size of the output layer obtained from the CNN.

**Figure 6:**
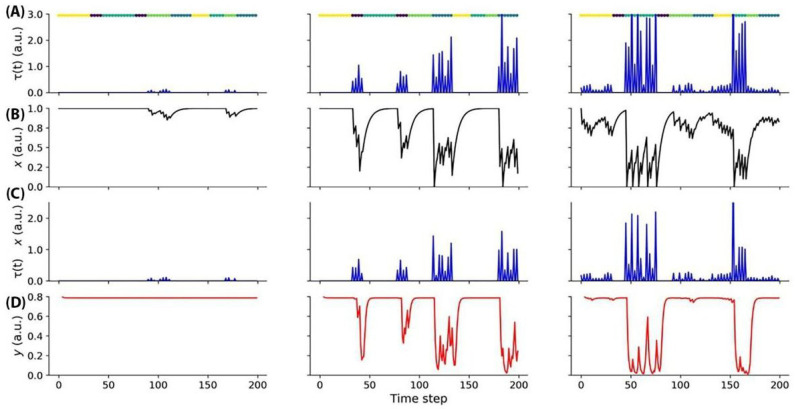
(**A**) The figure illustrates the input activity during the change detection task. Each image was presented for a duration of 250 ms (one time step), followed by a 500 ms (two time steps) gray screen. In the left block, weak responses to a single image are observed. The central block shows responses to two images, with one image eliciting a stronger response than the other. The right block gradually responds to four images. The time of image presentation is indicated by color-coded segments displayed above each plot. (**B**) The synaptic efficacy (depression) of the units depicted in panel A undergoes changes based on the received input. (**C**) The input activity of the units shown in panel A is modulated by short-term activity. (**D**) The dynamics of gliotransmitters.

Our model was evaluated in the context of the change detection task (Figure 2). The input activity provided to the model was sparse, aligning with the observed responses in a comprehensive survey conducted on the mouse visual cortex at a large scale (Figure 6).

Figure 6A depicts the activity patterns observed during the change detection task in response to the input stimuli. Each image in the sequence was presented for a duration of 250 ms, which corresponds to a single time step, followed by a 500 ms interval of a gray screen, spanning two time steps. In the left block of Figure 6A, the responses to a single image are relatively weak, indicating lower levels of neural activity. Moving to the central block, responses to two images are illustrated, with one of the images evoking a stronger response compared to the other. This discrepancy in response strengths signifies the neural discrimination between the presented stimuli. The right block of Figure 6A showcases a gradual increase in responses to four images, suggesting a progressive accumulation of neural activity over time. This gradual response pattern indicates the integration of information from multiple image presentations. To facilitate interpretation, color-coded segments displayed above each plot indicate the specific time points of image presentation, aiding in the visualization of the temporal dynamics of neural activity. By examining these activity patterns, we can gain insights into the temporal processing and encoding of visual information within the neural network involved in the change detection task.

The units depicted in panel A exhibit dynamic changes in synaptic efficacy, specifically through a process of synaptic depression, which is influenced by the input they receive (Figure 6B). These changes in synaptic efficacy are crucial for shaping the overall functioning of the neural network. As the units receive input, their synaptic connections undergo modifications, leading to the attenuation or reduction in the strength of synaptic transmission. This process of synaptic depression allows for the network to adapt to varying levels of input and maintain stability by preventing excessive neural activity. By dynamically adjusting synaptic efficacy, the network can effectively regulate the flow of information and optimize its response to the incoming stimuli. Understanding the mechanisms underlying synaptic depression provides valuable insights into the neural circuit’s ability to process and encode information, ultimately contributing to our understanding of neural computation. The activity of the units displayed in panel A is subject to modulation by short-term activity dynamics (Figure 6C). Short-term activity refers to the transient changes in neural firing rates and synaptic efficacy that occur over relatively brief time intervals. In the context of the depicted units, the input activity they receive is influenced and regulated by these short-term activity dynamics. By incorporating short-term activity modulation, the units are capable of flexibly adjusting their responsiveness to incoming input, allowing for dynamic processing and adaptation to changing environmental conditions. This modulation enhances the network’s ability to encode and integrate information, enabling more nuanced and context-dependent computations. The dynamics of gliotransmitters refers to the temporal changes and interactions of signaling molecules released (Figure 6D).

### 3.2. Learning Process

Algorithm 2 trains a convolutional neural network model on the CIFAR-10 dataset. It utilizes the stochastic gradient descent (SGD) algorithm to optimize the model parameters. The model comprises several convolutional layers, pooling layers, and fully connected layers. Training is conducted using the cross-entropy loss function.
**Algorithm 2** Learning algorithm for CNN model.**Input:** a grayscale image, represented as a single-channel image.**Parameter:** N_epochs, Batch_size, SGD optimizer, Cross-entropy loss function.**Output:** the probabilities of the image belonging to each of the classes.1.Initialize the neural network with a specific architecture (layers and connections) and initial weights.2.Load the data and set up the training parameters.3.**for** in N_epochs **do**4.      Set the neural network in training mode.5.      **for** Batch_size in training dataset **do**6.          Transfer the data and target values to the device (GPU or CPU) based on the settings.7.          Reset the gradients of the optimizer.8.          Perform a forward pass through the model to obtain the output values.9.          Calculate the loss function between the predicted and target values.10.           Perform backpropagation of gradients to compute them for each model parameter.11.           Update the model weights using the SGD optimizer.12.     **end for**13.**end for**14.Finish the algorithm execution.

Algorithm 1 presents the implementation of a training algorithm for a neural network. The algorithm incorporates the definition of the dynamic synapse model class, which is a neural network model that incorporates the dynamics of STPNet, astrocytic regulation, and spike generation. Additionally, the algorithm calculates the d-prime index.

### 3.3. Experimental Simulation

**Figure 7:**
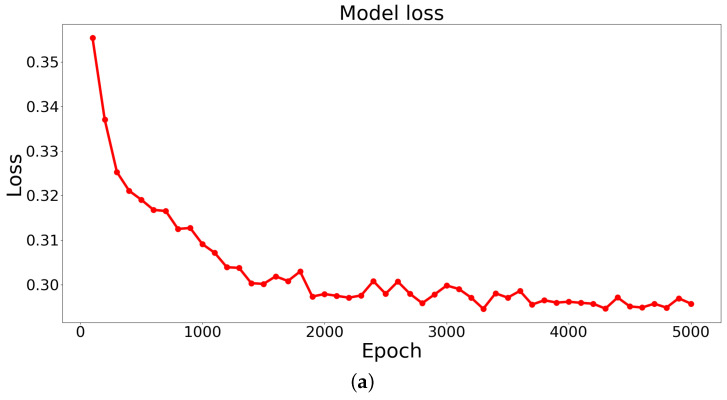

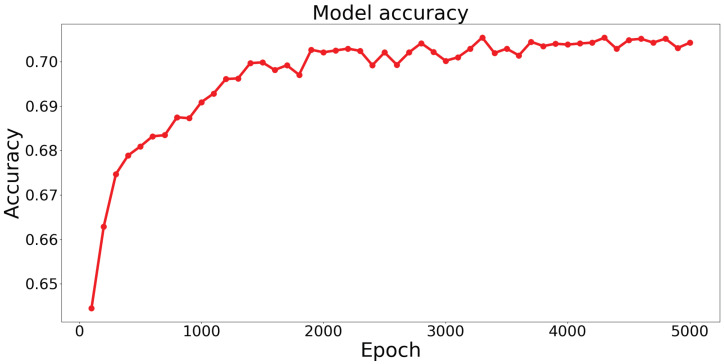
The graphs display the model’s (**a**) loss (error dependence on the number of epochs) and (**b**) accuracy (accuracy dependence on the number of epochs).

Based on the results obtained from training the STPANet model, accuracy and error graphs were generated (Figure 7).

From these graphs, the following conclusions can be drawn:

- Overtraining and undertraining are not observed.
- The optimal value of error and accuracy is reached at about 4000 epochs.

After around 4000 epochs, the model reached its peak performance, achieving the optimal balance between error reduction and accuracy improvement. This implies that further training iterations did not significantly contribute to enhancing the model’s performance.

The identification of this optimal point is crucial, as it allows for the selection of an appropriate stopping criterion during the training process, preventing unnecessary computational costs and saving resources. Additionally, it highlights the importance of monitoring the training progression and evaluating the model’s performance at different epochs to determine the point of convergence and achieve the desired level of accuracy.

**Figure 8:**
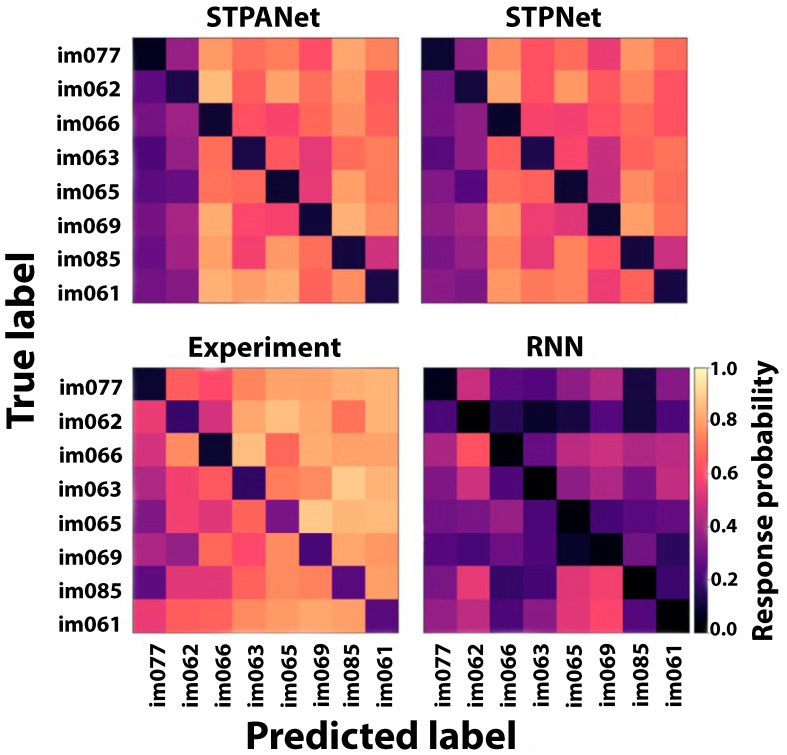
A confusion matrix is presented, which indicates the probability of responding to each of the possible 64 image transitions during the task. In the matrix, a value of 1 indicates that the image has not been shown before, while a value of 0 signifies that the image has already been shown.

Furthermore, in addition to generating plots for model comparison, we present the mean response probability matrices that illustrate the likelihood of a response for each of the 64 potential image transitions during task execution. The STPANet response probability matrix reveals an asymmetry in image detectability, which is also partially captured by the STPNet model but remains unaccounted for by the RNN model (Figure 8). The experimental data were obtained from the study by Hu et al. [32].

The confusion matrix serves as a valuable tool for evaluating the model’s performance in correctly identifying and distinguishing between different image transitions. This information aids in understanding the model’s capacity to learn and remember the visual patterns associated with specific image transitions. The inclusion of the confusion matrix allows for a comprehensive analysis of the model’s performance, facilitating the identification of potential areas of improvement and informing further training strategies.

After constructing confusion matrices to compare the presented models with the experiment, the asymmetry of the matrices was calculated for each model using Equation (11) (Table 1).

Based on the obtained values (Table 1), it can be observed that the STPANet model exhibits the strongest correlation with the experiment.

The calculated d-prime metric using Equation (6) for the three types of models is presented in Table 2 below:

The purpose of employing this metric was to demonstrate better classification accuracy achieved by our neural network architecture, which incorporates astrocytic modulation, for both familiar and novel images.

By utilizing this metric, we aimed to showcase the enhanced performance of our model in accurately categorizing not only images that were previously encountered but also those that were novel and unseen during the training phase. This ability to effectively generalize and classify unfamiliar images is a significant advantage of our neural network architecture.

The integration of astrocytic modulation within our architecture offers additional regulatory mechanisms that contribute to improved classification accuracy. The astrocytic modulation allows for dynamic adjustments of synaptic strength and facilitates efficient information processing and discrimination between different image categories.

## 4. Discussion

Based on analyzing neural and behavioral data related to the visual change detection problem [32], we constructed hybrid neuron network models targeting the implementation of the short-term memory features. Specifically, these models were utilized to test predictions of various short-term memory mechanisms. Our findings verified that adaptation mechanisms, rather than sustained activity, were employed for detecting changes in natural images. Additionally, we eventually demonstrated that accounting for the astrocytic regulation in the dynamical synapses led to better performance in the implementation of image processing tasks.

Our models indicate that short-term plasticity may support short-term memory in early sensory cortex neural circuits, acting as the main memory source in the change detection task. Multiple lines of evidence support our proposed model, including behavioral responses, neural response adaptation, and responses to omitted image presentations. Image repetition causes synapse adaptation, reducing information about image identity. Presentation of a change image activates a new set of input units, influencing hidden unit responses and facilitating image change decoding. Plasticity with astrocyte modulation acts as a temporal filter, enabling comparison of repeated and novel stimuli. Behavioral asymmetry results from different saliency levels, impacting stimulus processing. Models without bottom-up attention lack this behavior. While models with persistent neural activity can solve the task, they are less consistent with observed data. Recurrent neural networks tend to show symmetric responses, requiring further investigation. Depressing synapses on sensory input neurons may sufficiently capture neural dynamics in early sensory cortex for the change detection task. Causal optogenetic perturbations are needed to confirm our results.

In the context of the proposed approach, which involves the integration of an artificial neural network and a dynamic model, which can be used for constructing a spiking neural network, it is pertinent to delve into the distinctions between an artificial neural network and a spiking neural network. Spiking neural networks (SNNs) are distinctive due to their utilization of spiking neuron models, which transmit information through discrete spikes. These spikes carry temporal information and are well-suited for tasks involving precise timing, such as event-based vision. SNNs often employ spike-timing-dependent plasticity (STDP) for learning, capturing biological learning mechanisms [45,46,47].

On the other hand, artificial neural networks (ANNs), particularly convolutional neural networks (CNNs), are widely used for various image tasks such as image classification, object detection, and image segmentation. ANNs rely on continuous activation functions and gradient-based optimization methods for training [24]. This allows them to capture complex features and patterns within images, but they might lack the temporal precision inherent in SNNs.

While SNNs offer advantages in handling temporal information and energy efficiency through sparse spiking, they may require specialized hardware for efficient computation [48]. ANNs, on the other hand, are more established and practical due to their familiarity and widespread adoption. Furthermore, approaches are under development regarding the utilization of supervised, biologically plausible perceptron-based learning for spiking neural networks (SNNs). These approaches aim to construct deep SNNs and create hybrid models that combine convolutional neural networks (CNNs) with spiking neural networks [45].

## 5. Conclusions

Short-term memory, also known as working memory, is an essential cognitive process that involves temporarily storing and manipulating information. It plays a crucial role in attention, learning, problem-solving, and decision-making. This study presents a new hybrid model for short-term memory that combines short-term synaptic plasticity, astrocytic modulation of synaptic transmission, and a convolutional neural network. By comparing it with a recurrent neural network, the research demonstrates that the proposed model offers improved efficiency in accurately representing short-term memory.

Currently, convolutional neural networks are extensively utilized and, specifically, are actively employed in analyzing medical data related to socially significant diseases [26,49]. The suggested architecture enables the incorporation of a short-term memory effect into a convolutional neural network, thereby enhancing the capabilities of these architectures.

A possible future research direction could involve incorporating the regulation of synaptic transmission by the brain’s extracellular matrix into the model. Experimental and model studies suggest that this regulation can influence processes associated with memory and neural network activity [50,51,52].

## Figures and Tables

**Table 1:** The table shows the asymmetry metrics of the matrices.

Model	Mean	Std
Experiment	−0.066	0.054
RNN	−0.201	0.356
STPNet	−0.034	0.09
**STPANet**	**−0.041**	0.09

**Table 2:** The average d-prime metric values, accompanied by their respective standard deviations, were computed for three models: RNN, STPNet, and STPANet.

Model	Mean	Std
RNN	1.33	0.578
STPNet	1.47	0.127
**STPANet**	**1.53**	0.125

## Data Availability

The data that support the findings of this study are available from the corresponding author upon reasonable request.

## Abbreviations

The following abbreviations are used in this manuscript:

STPNet	Convolutional neural network with short-term plasticity
STPANet	Convolutional neural network with short-term plasticity and astrocyte modulation
RNN	Recurrent neural network
CNN	Convolutional neural network
